# Poly[[bis­(nitrato-κ*O*)bis­(μ_4_-pyridine-4-carboxyl­ato)tetra­kis­(μ_3_-pyridine-4-carboxyl­ato)octa­silver(I)] hemihydrate]

**DOI:** 10.1107/S1600536811028261

**Published:** 2011-07-23

**Authors:** Zhao-Hui Meng

**Affiliations:** a College of Chemistry and Pharmacy Engineering, Nanyang Normal University, Nanyang 473061, People’s Republic of China

## Abstract

In the title coordination polymer, {[Ag_8_(C_6_H_4_NO_2_)_6_(NO_3_)_2_]·0.5H_2_O}_*n*_, two Ag^I^ ions are two-coordinate within an AgN_2_ set and six are three-coordinate within AgN_2_O and AgO_3_ sets. The Ag—N and Ag—O distances are in the ranges 2.150 (5)–2.198 (5) and 2.142 (4)–2.702 (5) Å, respectively. A two-dimensional coordination network is formed parallel to (100). The O atom of the disordered solvent water mol­ecule is located on an inversion center.

## Related literature

For examples of silver(I) coordination compounds containing isonicotinic acid, see: Du & Zhao (2004 ▶); Jaber *et al.* (1994 ▶); Yang *et al.* (2004 ▶).
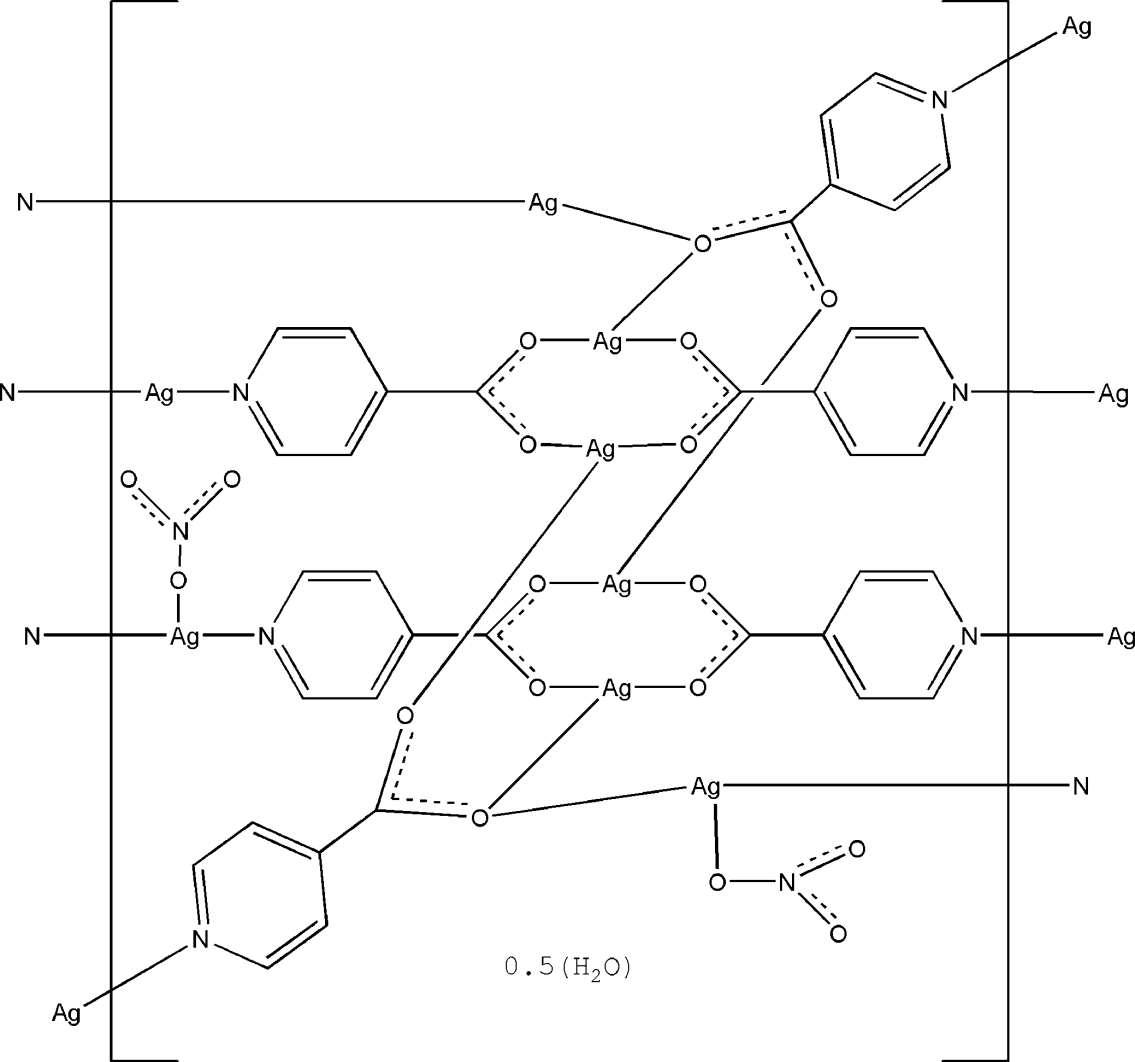


## Experimental

### 

#### Crystal data

[Ag_8_(C_6_H_4_NO_2_)_6_(NO_3_)_2_]·0.5H_2_O
*M* *_r_* = 1728.59
Monoclinic, 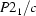

*a* = 18.006 (4) Å
*b* = 18.255 (4) Å
*c* = 13.166 (3) Å
β = 104.017 (4)°
*V* = 4199.0 (17) Å^3^
*Z* = 4
Mo *K*α radiation
μ = 3.74 mm^−1^
*T* = 296 K
0.23 × 0.17 × 0.15 mm

#### Data collection

Bruker APEXII CCD diffractometer
Absorption correction: multi-scan (*SADABS*; Bruker, 2008 ▶) *T*_min_ = 0.480, *T*_max_ = 0.604
21106 measured reflections
7391 independent reflections
6057 reflections with *I* > 2σ(*I*)
*R*_int_ = 0.040

#### Refinement

*R*[*F* ^2^ > 2σ(*F* ^2^)] = 0.041
*wR*(*F* ^2^) = 0.106
*S* = 1.01
7391 reflections
637 parameters
H-atom parameters constrained
Δρ_max_ = 1.52 e Å^−3^
Δρ_min_ = −1.68 e Å^−3^

### 

Data collection: *APEX2* (Bruker, 2008 ▶); cell refinement: *SAINT* (Bruker, 2008 ▶); data reduction: *SAINT*; program(s) used to solve structure: *SHELXS97* (Sheldrick, 2008 ▶); program(s) used to refine structure: *SHELXL97* (Sheldrick, 2008 ▶); molecular graphics: *SHELXTL* (Sheldrick, 2008 ▶); software used to prepare material for publication: *SHELXTL*.

## Supplementary Material

Crystal structure: contains datablock(s) I, global. DOI: 10.1107/S1600536811028261/gk2394sup1.cif

Structure factors: contains datablock(s) I. DOI: 10.1107/S1600536811028261/gk2394Isup2.hkl

Additional supplementary materials:  crystallographic information; 3D view; checkCIF report

## Figures and Tables

**Fig. 1.**
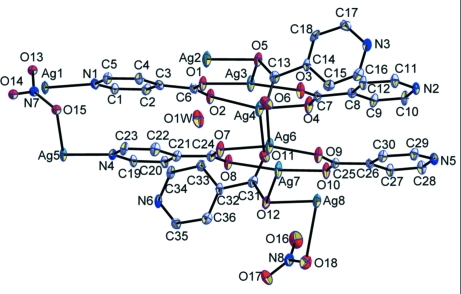
View of the asymmetric unit of the title compound with displacement ellipsoids drawn at the 30% probability level. Hadrogen atoms are omitted for clarity.

**Fig. 2.**
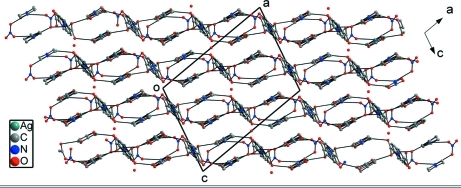
Crystal packing viewed along the b axis (H atoms are omitted for clarity).

## supplementary crystallographic information

### Comment

Herein, using isonicotinic acid as bridging ligand, we report a two-dimensional coordination polymer {[Ag_8_(C_6_H_4_NO_2_)_6_(NO_3_)_2_].0.5H_2_O}_n_. The asymmetric unit of the title compound contains eight crystallographically independent Ag^I ^atoms, six C_6_H_4_NO_2 _ligands, two nitrate ligands and half water molecule (Fig. 1). In the structure of title compound, two Ag^I^ atoms are two-coordinate (Ag1, Ag2) and six are three-coordinate. The bond lengths Ag—N and Ag—O range from 2.150 (5) to 2.198 (5) Å and 2.142 (4) to 2.702 (5)) Å, respectively. The two dimensional polymeric structure of the title coordination polymer is parallel to (1 0 0). The disordered water molecule that is located on inversion center connects *via* hydrogen bonds adjacent coordination polymers (Fig. 2). It is also in a short contact [2.832 (4) Å] to two Ag(2) atoms from the neighbouring polymers.

### Experimental

All chemicals were of reagent grade quality obtained from commercial sources and used without further purification. The title compound was synthesized from a mixture of AgNO_3_ (0.34 g, 2 mmol), C_6_H_5_NO_2_ (0.26 g, 1.2 mmol) and H_2_O (12 g, 667 mmol) in a molar ratio of 2: 1.2: 667 by hydrothermal reaction. The mixture was stirred for half an hour, and then transferred into a Teflon-lined stainless steel autoclave (50 ml) and treated at 160 °C for 3 days. After the mixture was slowly cooled to room temperature, colorless rod-shaped crystals were obtained.

### Refinement

The H atoms bonded to C were positioned geometrically and refined using a riding model, with C—H = 0.93 Å and with *U*_iso_(H) = 1.2 times *U*_eq_(C). The H atoms of O1W were not located due to disorder of water molecule.

### Crystal data

**Table:**

[Ag_8_(C_6_H_4_NO_2_)_6_(NO_3_)_2_]·0.5H_2_O	*F*(000) = 3280
*M**_r_* = 1728.59	*D*_x_ = 2.733 Mg m^−^^3^
Monoclinic, *P*2_1_/*c*	Mo *K*α radiation, λ = 0.71073 Å
Hall symbol: -P 2ybc	Cell parameters from 7885 reflections
*a* = 18.006 (4) Å	θ = 2.5–28.3°
*b* = 18.255 (4) Å	µ = 3.74 mm^−^^1^
*c* = 13.166 (3) Å	*T* = 296 K
β = 104.017 (4)°	Rod, colorless
*V* = 4199.0 (17) Å^3^	0.23 × 0.17 × 0.15 mm
*Z* = 4	

### Data collection

**Table:**

Bruker APEXII CCD diffractometer	7391 independent reflections
Radiation source: fine-focus sealed tube	6057 reflections with *I* > 2σ(*I*)
graphite	*R*_int_ = 0.040
φ and ω scans	θ_max_ = 25.0°, θ_min_ = 1.2°
Absorption correction: multi-scan (*SADABS*; Bruker, 2008)	*h* = −21→20
*T*_min_ = 0.480, *T*_max_ = 0.604	*k* = −15→21
21106 measured reflections	*l* = −15→15

### Refinement

**Table:**

Refinement on *F*^2^	Primary atom site location: structure-invariant direct methods
Least-squares matrix: full	Secondary atom site location: difference Fourier map
*R*[*F*^2^ > 2σ(*F*^2^)] = 0.041	Hydrogen site location: inferred from neighbouring sites
*wR*(*F*^2^) = 0.106	H-atom parameters constrained
*S* = 1.01	*w* = 1/[σ^2^(*F*_o_^2^) + (0.0468*P*)^2^ + 11.8118*P*] where *P* = (*F*_o_^2^ + 2*F*_c_^2^)/3
7391 reflections	(Δ/σ)_max_ = 0.001
637 parameters	Δρ_max_ = 1.52 e Å^−^^3^
0 restraints	Δρ_min_ = −1.68 e Å^−^^3^

### Special details

**Table:**

Geometry. All e.s.d.'s (except the e.s.d. in the dihedral angle between two l.s. planes) are estimated using the full covariance matrix. The cell e.s.d.'s are taken into account individually in the estimation of e.s.d.'s in distances, angles and torsion angles; correlations between e.s.d.'s in cell parameters are only used when they are defined by crystal symmetry. An approximate (isotropic) treatment of cell e.s.d.'s is used for estimating e.s.d.'s involving l.s. planes.

### Fractional atomic coordinates and isotropic or equivalent isotropic displacement parameters (Å^2^)

**Table:**

	*x*	*y*	*z*	*U*_iso_*/*U*_eq_	
Ag1	0.76442 (3)	0.09112 (2)	0.37019 (4)	0.04168 (14)	
Ag2	0.52204 (4)	0.51064 (3)	0.29474 (6)	0.0731 (2)	
Ag3	0.69514 (3)	0.59092 (2)	0.36445 (5)	0.04876 (15)	
Ag4	0.85616 (3)	0.58930 (2)	0.43687 (5)	0.05365 (16)	
Ag5	0.73415 (3)	0.14657 (2)	0.60134 (4)	0.04101 (13)	
Ag6	0.65019 (3)	0.64893 (2)	0.58104 (5)	0.05103 (16)	
Ag7	0.81026 (3)	0.64803 (2)	0.65006 (5)	0.05424 (16)	
Ag8	0.96867 (3)	0.71827 (3)	0.74350 (4)	0.04974 (16)	
C1	0.8346 (3)	0.2460 (3)	0.3692 (4)	0.0321 (12)	
H1A	0.8795	0.2193	0.3761	0.039*	
C2	0.8397 (3)	0.3210 (3)	0.3714 (4)	0.0346 (13)	
H2A	0.8868	0.3442	0.3791	0.042*	
C3	0.7728 (3)	0.3617 (3)	0.3619 (4)	0.0308 (12)	
C4	0.7046 (3)	0.3241 (3)	0.3456 (4)	0.0333 (12)	
H4A	0.6587	0.3497	0.3355	0.040*	
C5	0.7040 (3)	0.2483 (3)	0.3441 (4)	0.0351 (13)	
H5A	0.6574	0.2239	0.3335	0.042*	
C6	0.7750 (3)	0.4435 (3)	0.3741 (4)	0.0321 (12)	
C7	0.7793 (3)	0.7381 (3)	0.4018 (4)	0.0325 (12)	
C8	0.7765 (3)	0.8202 (3)	0.3910 (4)	0.0290 (12)	
C9	0.8430 (3)	0.8631 (3)	0.4116 (5)	0.0364 (13)	
H9A	0.8909	0.8410	0.4304	0.044*	
C10	0.8370 (3)	0.9371 (3)	0.4040 (5)	0.0378 (14)	
H10A	0.8819	0.9645	0.4182	0.045*	
C11	0.7065 (3)	0.9316 (3)	0.3558 (5)	0.0386 (14)	
H11A	0.6592	0.9549	0.3354	0.046*	
C12	0.7076 (3)	0.8572 (3)	0.3625 (5)	0.0376 (14)	
H12A	0.6618	0.8312	0.3478	0.045*	
C13	0.5378 (3)	0.6716 (3)	0.3558 (5)	0.0345 (13)	
C14	0.5225 (3)	0.7494 (3)	0.3154 (4)	0.0314 (12)	
C15	0.5159 (3)	0.8054 (3)	0.3857 (5)	0.0378 (13)	
H15A	0.5198	0.7948	0.4559	0.045*	
C16	0.5038 (3)	0.8759 (3)	0.3499 (5)	0.0396 (14)	
H16A	0.5005	0.9126	0.3977	0.047*	
C17	0.5029 (3)	0.8405 (3)	0.1828 (5)	0.0385 (13)	
H17A	0.4980	0.8519	0.1127	0.046*	
C18	0.5172 (3)	0.7664 (3)	0.2162 (4)	0.0322 (12)	
H18A	0.5227	0.7303	0.1687	0.039*	
C19	0.7952 (3)	0.3043 (3)	0.6191 (4)	0.0312 (12)	
H19A	0.8419	0.2802	0.6290	0.037*	
C20	0.7946 (3)	0.3794 (3)	0.6228 (4)	0.0329 (12)	
H20A	0.8405	0.4051	0.6352	0.039*	
C21	0.7261 (3)	0.4178 (3)	0.6082 (4)	0.0304 (12)	
C22	0.6593 (3)	0.3759 (3)	0.5905 (5)	0.0374 (13)	
H22A	0.6119	0.3986	0.5810	0.045*	
C23	0.6643 (3)	0.3014 (3)	0.5873 (5)	0.0372 (13)	
H23A	0.6190	0.2746	0.5744	0.045*	
C24	0.7258 (3)	0.4997 (3)	0.6084 (4)	0.0321 (12)	
C25	0.7286 (3)	0.7952 (3)	0.6344 (4)	0.0314 (12)	
C26	0.7287 (3)	0.8778 (3)	0.6362 (4)	0.0272 (11)	
C27	0.7969 (3)	0.9165 (3)	0.6531 (5)	0.0336 (13)	
H27A	0.8433	0.8916	0.6706	0.040*	
C28	0.7958 (3)	0.9911 (3)	0.6442 (5)	0.0368 (13)	
H28A	0.8422	1.0159	0.6549	0.044*	
C29	0.6649 (3)	0.9926 (3)	0.6096 (4)	0.0350 (13)	
H29A	0.6195	1.0190	0.5965	0.042*	
C30	0.6611 (3)	0.9183 (3)	0.6164 (4)	0.0325 (12)	
H30A	0.6141	0.8950	0.6081	0.039*	
C31	0.9641 (3)	0.5647 (3)	0.6772 (5)	0.0326 (12)	
C32	0.9812 (3)	0.4839 (3)	0.7003 (4)	0.0303 (12)	
C33	0.9862 (3)	0.4364 (3)	0.6201 (4)	0.0334 (12)	
H33A	0.9802	0.4538	0.5522	0.040*	
C34	1.0004 (3)	0.3628 (3)	0.6419 (5)	0.0371 (13)	
H34A	1.0040	0.3314	0.5876	0.045*	
C35	1.0030 (3)	0.3805 (3)	0.8162 (5)	0.0388 (14)	
H35A	1.0074	0.3617	0.8830	0.047*	
C36	0.9906 (3)	0.4552 (3)	0.8002 (5)	0.0366 (13)	
H36A	0.9886	0.4858	0.8560	0.044*	
N1	0.7685 (3)	0.2089 (2)	0.3576 (4)	0.0337 (11)	
N2	0.7698 (2)	0.9733 (2)	0.3771 (4)	0.0304 (10)	
N3	0.4963 (3)	0.8948 (2)	0.2501 (4)	0.0372 (11)	
N4	0.7306 (2)	0.2645 (2)	0.6016 (3)	0.0297 (10)	
N5	0.7309 (3)	1.0298 (3)	0.6206 (4)	0.0352 (11)	
N6	1.0091 (3)	0.3347 (2)	0.7392 (4)	0.0356 (11)	
N7	0.4240 (3)	0.5945 (2)	0.1017 (4)	0.0366 (11)	
N8	1.0749 (3)	0.6422 (3)	0.9356 (4)	0.0395 (12)	
O1	0.7108 (2)	0.4747 (2)	0.3625 (3)	0.0425 (10)	
O2	0.8390 (2)	0.4738 (2)	0.3961 (4)	0.0455 (11)	
O3	0.7162 (2)	0.7071 (2)	0.3867 (4)	0.0466 (11)	
O4	0.8434 (2)	0.7086 (2)	0.4260 (4)	0.0538 (12)	
O5	0.5583 (2)	0.6274 (2)	0.2934 (4)	0.0431 (10)	
O6	0.5296 (2)	0.6566 (2)	0.4441 (4)	0.0494 (11)	
O7	0.6614 (2)	0.5305 (2)	0.5919 (4)	0.0490 (11)	
O8	0.7888 (2)	0.5311 (2)	0.6248 (4)	0.0428 (10)	
O9	0.6638 (2)	0.7644 (2)	0.6112 (4)	0.0455 (11)	
O10	0.7926 (2)	0.7648 (2)	0.6547 (4)	0.0465 (11)	
O11	0.9665 (3)	0.5894 (2)	0.5911 (3)	0.0460 (11)	
O12	0.9471 (2)	0.6010 (2)	0.7502 (3)	0.0421 (10)	
O13	0.3939 (3)	0.5843 (3)	0.1764 (4)	0.0555 (12)	
O14	0.4752 (3)	0.5534 (3)	0.0880 (4)	0.0564 (12)	
O15	0.4022 (3)	0.6465 (2)	0.0407 (4)	0.0558 (12)	
O16	1.1085 (3)	0.6510 (3)	0.8641 (5)	0.0647 (14)	
O17	1.0887 (3)	0.5885 (3)	0.9939 (4)	0.0659 (14)	
O18	1.0269 (2)	0.6890 (2)	0.9478 (4)	0.0518 (11)	
O1W	0.5000	0.5000	0.5000	0.065 (2)	

### Atomic displacement parameters (Å^2^)

**Table:**

	*U*^11^	*U*^22^	*U*^33^	*U*^12^	*U*^13^	*U*^23^
Ag1	0.0553 (3)	0.0149 (2)	0.0555 (3)	−0.00049 (18)	0.0148 (2)	0.00041 (19)
Ag2	0.0813 (4)	0.0253 (3)	0.0924 (5)	−0.0090 (3)	−0.0183 (3)	0.0034 (3)
Ag3	0.0480 (3)	0.0187 (2)	0.0799 (4)	0.00469 (19)	0.0163 (3)	0.0013 (2)
Ag4	0.0476 (3)	0.0209 (2)	0.0847 (4)	0.0022 (2)	0.0010 (3)	−0.0017 (2)
Ag5	0.0528 (3)	0.0155 (2)	0.0547 (3)	−0.00172 (18)	0.0130 (2)	0.00065 (18)
Ag6	0.0421 (3)	0.0197 (2)	0.0842 (4)	0.00300 (19)	0.0015 (3)	−0.0021 (2)
Ag7	0.0426 (3)	0.0182 (2)	0.0974 (4)	0.00448 (19)	0.0082 (3)	−0.0001 (2)
Ag8	0.0529 (3)	0.0203 (2)	0.0690 (3)	−0.00273 (19)	0.0011 (3)	−0.0019 (2)
C1	0.033 (3)	0.026 (3)	0.037 (3)	0.003 (2)	0.009 (2)	0.000 (2)
C2	0.033 (3)	0.030 (3)	0.040 (3)	0.001 (2)	0.008 (2)	−0.002 (3)
C3	0.037 (3)	0.024 (3)	0.030 (3)	0.001 (2)	0.005 (2)	0.004 (2)
C4	0.031 (3)	0.029 (3)	0.041 (3)	0.004 (2)	0.011 (2)	0.000 (2)
C5	0.032 (3)	0.025 (3)	0.046 (3)	−0.001 (2)	0.005 (3)	−0.002 (3)
C6	0.042 (3)	0.022 (3)	0.033 (3)	0.006 (2)	0.011 (2)	0.006 (2)
C7	0.039 (3)	0.017 (3)	0.040 (3)	0.004 (2)	0.007 (3)	0.000 (2)
C8	0.032 (3)	0.023 (3)	0.031 (3)	0.000 (2)	0.007 (2)	0.000 (2)
C9	0.026 (3)	0.026 (3)	0.057 (4)	0.000 (2)	0.010 (3)	−0.008 (3)
C10	0.032 (3)	0.021 (3)	0.064 (4)	−0.007 (2)	0.018 (3)	−0.003 (3)
C11	0.030 (3)	0.025 (3)	0.059 (4)	0.002 (2)	0.007 (3)	0.004 (3)
C12	0.026 (3)	0.025 (3)	0.059 (4)	−0.003 (2)	0.006 (3)	−0.002 (3)
C13	0.020 (3)	0.034 (3)	0.048 (4)	−0.005 (2)	0.004 (2)	0.003 (3)
C14	0.023 (3)	0.022 (3)	0.047 (3)	−0.005 (2)	0.005 (2)	−0.003 (2)
C15	0.038 (3)	0.038 (3)	0.041 (3)	−0.001 (3)	0.016 (3)	−0.003 (3)
C16	0.035 (3)	0.035 (3)	0.049 (4)	−0.002 (3)	0.011 (3)	−0.012 (3)
C17	0.036 (3)	0.038 (3)	0.041 (3)	−0.003 (3)	0.007 (3)	0.006 (3)
C18	0.024 (3)	0.033 (3)	0.042 (3)	−0.010 (2)	0.011 (2)	−0.016 (3)
C19	0.029 (3)	0.020 (3)	0.044 (3)	0.003 (2)	0.007 (2)	0.004 (2)
C20	0.026 (3)	0.027 (3)	0.044 (3)	−0.003 (2)	0.004 (2)	0.002 (2)
C21	0.030 (3)	0.026 (3)	0.035 (3)	−0.002 (2)	0.007 (2)	−0.005 (2)
C22	0.024 (3)	0.031 (3)	0.056 (4)	0.000 (2)	0.008 (3)	−0.004 (3)
C23	0.036 (3)	0.024 (3)	0.053 (4)	−0.010 (2)	0.014 (3)	−0.007 (3)
C24	0.031 (3)	0.025 (3)	0.039 (3)	0.001 (2)	0.007 (2)	−0.001 (2)
C25	0.041 (3)	0.021 (3)	0.033 (3)	0.002 (2)	0.010 (2)	0.000 (2)
C26	0.031 (3)	0.019 (3)	0.031 (3)	0.003 (2)	0.009 (2)	0.002 (2)
C27	0.028 (3)	0.025 (3)	0.048 (3)	0.005 (2)	0.009 (3)	0.000 (2)
C28	0.031 (3)	0.026 (3)	0.056 (4)	−0.001 (2)	0.014 (3)	0.002 (3)
C29	0.030 (3)	0.028 (3)	0.045 (3)	0.003 (2)	0.005 (3)	−0.002 (3)
C30	0.029 (3)	0.026 (3)	0.043 (3)	−0.001 (2)	0.009 (2)	−0.003 (2)
C31	0.021 (3)	0.031 (3)	0.043 (3)	−0.002 (2)	0.003 (2)	−0.005 (3)
C32	0.024 (3)	0.023 (3)	0.044 (3)	−0.003 (2)	0.006 (2)	−0.001 (2)
C33	0.034 (3)	0.030 (3)	0.038 (3)	−0.003 (2)	0.014 (2)	−0.002 (2)
C34	0.040 (3)	0.022 (3)	0.052 (4)	−0.004 (2)	0.015 (3)	−0.006 (3)
C35	0.039 (3)	0.033 (3)	0.041 (3)	0.000 (3)	0.006 (3)	0.008 (3)
C36	0.034 (3)	0.032 (3)	0.042 (3)	−0.001 (2)	0.007 (3)	−0.006 (3)
N1	0.045 (3)	0.021 (2)	0.037 (3)	0.004 (2)	0.014 (2)	0.003 (2)
N2	0.031 (2)	0.016 (2)	0.044 (3)	0.0005 (18)	0.011 (2)	0.0007 (19)
N3	0.034 (3)	0.024 (2)	0.051 (3)	−0.002 (2)	0.007 (2)	−0.002 (2)
N4	0.033 (2)	0.020 (2)	0.037 (2)	−0.0043 (19)	0.011 (2)	−0.0021 (19)
N5	0.040 (3)	0.022 (2)	0.044 (3)	0.001 (2)	0.013 (2)	0.000 (2)
N6	0.038 (3)	0.017 (2)	0.053 (3)	0.002 (2)	0.014 (2)	−0.002 (2)
N7	0.029 (2)	0.026 (3)	0.050 (3)	−0.001 (2)	0.000 (2)	−0.007 (2)
N8	0.031 (3)	0.033 (3)	0.051 (3)	−0.002 (2)	0.003 (2)	0.000 (2)
O1	0.041 (2)	0.0182 (19)	0.067 (3)	0.0049 (17)	0.011 (2)	−0.0009 (19)
O2	0.042 (2)	0.019 (2)	0.073 (3)	−0.0013 (18)	0.008 (2)	−0.002 (2)
O3	0.037 (2)	0.021 (2)	0.082 (3)	−0.0027 (18)	0.016 (2)	−0.001 (2)
O4	0.041 (2)	0.023 (2)	0.094 (4)	0.0031 (19)	0.010 (2)	−0.001 (2)
O5	0.041 (2)	0.020 (2)	0.065 (3)	−0.0001 (17)	0.008 (2)	−0.003 (2)
O6	0.042 (2)	0.053 (3)	0.053 (3)	0.003 (2)	0.011 (2)	0.018 (2)
O7	0.032 (2)	0.022 (2)	0.089 (3)	0.0034 (17)	0.009 (2)	−0.002 (2)
O8	0.033 (2)	0.020 (2)	0.073 (3)	−0.0020 (17)	0.008 (2)	−0.0052 (19)
O9	0.039 (2)	0.020 (2)	0.078 (3)	−0.0022 (17)	0.014 (2)	−0.004 (2)
O10	0.036 (2)	0.019 (2)	0.083 (3)	0.0057 (17)	0.012 (2)	0.002 (2)
O11	0.061 (3)	0.032 (2)	0.042 (2)	0.004 (2)	0.007 (2)	0.0069 (19)
O12	0.056 (3)	0.018 (2)	0.054 (3)	0.0039 (18)	0.015 (2)	−0.0057 (18)
O13	0.042 (3)	0.063 (3)	0.068 (3)	−0.002 (2)	0.025 (2)	0.007 (3)
O14	0.048 (3)	0.050 (3)	0.070 (3)	0.016 (2)	0.012 (2)	−0.006 (2)
O15	0.063 (3)	0.041 (3)	0.059 (3)	0.009 (2)	0.007 (2)	0.010 (2)
O16	0.045 (3)	0.063 (3)	0.092 (4)	−0.002 (2)	0.028 (3)	0.007 (3)
O17	0.085 (4)	0.041 (3)	0.067 (3)	0.013 (3)	0.010 (3)	0.018 (3)
O18	0.043 (2)	0.045 (3)	0.063 (3)	0.013 (2)	0.003 (2)	−0.007 (2)
O1W	0.044 (4)	0.044 (4)	0.101 (6)	−0.004 (3)	0.004 (4)	0.013 (4)

### Geometric parameters (Å, °)

**Table:**

Ag1—N2^i^	2.154 (4)	C14—C15	1.404 (8)
Ag1—N1	2.159 (4)	C15—C16	1.371 (8)
Ag1—Ag5	3.3746 (10)	C15—H15A	0.9300
Ag2—N3^ii^	2.198 (5)	C16—N3	1.333 (8)
Ag2—O5	2.230 (4)	C16—H16A	0.9300
Ag2—Ag3	3.3643 (10)	C17—N3	1.354 (8)
Ag3—O1	2.142 (4)	C17—C18	1.427 (8)
Ag3—O3	2.161 (4)	C17—H17A	0.9300
Ag3—O5	2.504 (4)	C18—H18A	0.9300
Ag3—Ag4	2.8244 (9)	C19—N4	1.345 (7)
Ag3—Ag6	3.3207 (10)	C19—C20	1.373 (7)
Ag4—O2	2.179 (4)	C19—H19A	0.9300
Ag4—O4	2.191 (4)	C20—C21	1.390 (7)
Ag4—O11	2.472 (4)	C20—H20A	0.9300
Ag4—Ag7	3.2916 (11)	C21—C22	1.396 (8)
Ag5—N5^i^	2.150 (5)	C21—C24	1.496 (8)
Ag5—N4	2.154 (4)	C22—C23	1.364 (8)
Ag5—O15^ii^	2.702 (5)	C22—H22A	0.9300
Ag6—O9	2.149 (4)	C23—N4	1.344 (7)
Ag6—O7	2.173 (4)	C23—H23A	0.9300
Ag6—O6	2.468 (4)	C24—O8	1.242 (6)
Ag6—Ag7	2.8043 (9)	C24—O7	1.260 (6)
Ag7—O10	2.158 (4)	C25—O10	1.248 (7)
Ag7—O8	2.180 (4)	C25—O9	1.265 (7)
Ag7—O12	2.641 (4)	C25—C26	1.508 (7)
Ag7—Ag8	3.0973 (9)	C26—C27	1.386 (7)
Ag8—N6^iii^	2.164 (4)	C26—C30	1.395 (7)
Ag8—O12	2.182 (4)	C27—C28	1.367 (8)
C1—N1	1.347 (7)	C27—H27A	0.9300
C1—C2	1.371 (8)	C28—N5	1.337 (7)
C1—H1A	0.9300	C28—H28A	0.9300
C2—C3	1.395 (8)	C29—N5	1.345 (7)
C2—H2A	0.9300	C29—C30	1.361 (8)
C3—C4	1.379 (8)	C29—H29A	0.9300
C3—C6	1.500 (8)	C30—H30A	0.9300
C4—C5	1.384 (8)	C31—O11	1.231 (7)
C4—H4A	0.9300	C31—O12	1.265 (7)
C5—N1	1.339 (7)	C31—C32	1.522 (7)
C5—H5A	0.9300	C32—C36	1.387 (8)
C6—O2	1.248 (7)	C32—C33	1.387 (8)
C6—O1	1.265 (7)	C33—C34	1.385 (8)
C7—O3	1.242 (7)	C33—H33A	0.9300
C7—O4	1.244 (7)	C34—N6	1.354 (8)
C7—C8	1.505 (7)	C34—H34A	0.9300
C8—C12	1.383 (7)	C35—N6	1.339 (7)
C8—C9	1.400 (7)	C35—C36	1.390 (8)
C9—C10	1.359 (8)	C35—H35A	0.9300
C9—H9A	0.9300	C36—H36A	0.9300
C10—N2	1.348 (7)	N2—Ag1^iv^	2.154 (4)
C10—H10A	0.9300	N3—Ag2^v^	2.198 (5)
C11—N2	1.342 (7)	N5—Ag5^iv^	2.150 (5)
C11—C12	1.361 (8)	N6—Ag8^vi^	2.164 (4)
C11—H11A	0.9300	N7—O14	1.237 (6)
C12—H12A	0.9300	N7—O15	1.244 (6)
C13—O6	1.238 (7)	N7—O13	1.246 (6)
C13—O5	1.269 (7)	N8—O17	1.233 (6)
C13—C14	1.517 (8)	N8—O16	1.246 (7)
C14—C18	1.323 (8)	N8—O18	1.253 (6)
			
N2^i^—Ag1—N1	174.66 (17)	N3—C16—C15	123.5 (5)
N2^i^—Ag1—Ag5	106.07 (12)	N3—C16—H16A	118.3
N1—Ag1—Ag5	77.67 (12)	C15—C16—H16A	118.3
N3^ii^—Ag2—O5	160.41 (18)	N3—C17—C18	122.1 (5)
N3^ii^—Ag2—Ag3	124.16 (13)	N3—C17—H17A	119.0
O5—Ag2—Ag3	48.09 (10)	C18—C17—H17A	119.0
O1—Ag3—O3	162.63 (16)	C14—C18—C17	119.4 (5)
O1—Ag3—O5	112.27 (14)	C14—C18—H18A	120.3
O3—Ag3—O5	85.10 (14)	C17—C18—H18A	120.3
O1—Ag3—Ag4	82.33 (11)	N4—C19—C20	122.1 (5)
O3—Ag3—Ag4	80.35 (11)	N4—C19—H19A	119.0
O5—Ag3—Ag4	164.91 (9)	C20—C19—H19A	119.0
O1—Ag3—Ag6	112.72 (12)	C19—C20—C21	120.9 (5)
O3—Ag3—Ag6	69.02 (12)	C19—C20—H20A	119.6
O5—Ag3—Ag6	79.26 (10)	C21—C20—H20A	119.6
Ag4—Ag3—Ag6	99.04 (2)	C20—C21—C22	116.6 (5)
O1—Ag3—Ag2	71.38 (11)	C20—C21—C24	120.4 (5)
O3—Ag3—Ag2	125.80 (11)	C22—C21—C24	123.0 (5)
O5—Ag3—Ag2	41.52 (9)	C23—C22—C21	119.4 (5)
Ag4—Ag3—Ag2	153.50 (2)	C23—C22—H22A	120.3
Ag6—Ag3—Ag2	88.33 (2)	C21—C22—H22A	120.3
O2—Ag4—O4	159.94 (18)	N4—C23—C22	124.0 (5)
O2—Ag4—O11	103.94 (15)	N4—C23—H23A	118.0
O4—Ag4—O11	95.94 (15)	C22—C23—H23A	118.0
O2—Ag4—Ag3	81.62 (11)	O8—C24—O7	126.0 (5)
O4—Ag4—Ag3	83.38 (11)	O8—C24—C21	117.3 (5)
O11—Ag4—Ag3	146.30 (11)	O7—C24—C21	116.7 (5)
O2—Ag4—Ag7	118.37 (13)	O10—C25—O9	127.1 (5)
O4—Ag4—Ag7	71.82 (14)	O10—C25—C26	116.4 (5)
O11—Ag4—Ag7	67.49 (11)	O9—C25—C26	116.5 (5)
Ag3—Ag4—Ag7	80.52 (2)	C27—C26—C30	117.3 (5)
N5^i^—Ag5—N4	171.68 (17)	C27—C26—C25	120.6 (5)
N5^i^—Ag5—Ag1	79.87 (13)	C30—C26—C25	122.0 (5)
N4—Ag5—Ag1	108.24 (12)	C28—C27—C26	120.0 (5)
O9—Ag6—O7	163.67 (17)	C28—C27—H27A	120.0
O9—Ag6—O6	96.89 (16)	C26—C27—H27A	120.0
O7—Ag6—O6	98.96 (16)	N5—C28—C27	122.6 (5)
O9—Ag6—Ag7	83.33 (11)	N5—C28—H28A	118.7
O7—Ag6—Ag7	84.33 (11)	C27—C28—H28A	118.7
O6—Ag6—Ag7	152.96 (11)	N5—C29—C30	123.6 (5)
O9—Ag6—Ag3	115.54 (12)	N5—C29—H29A	118.2
O7—Ag6—Ag3	72.63 (13)	C30—C29—H29A	118.2
O6—Ag6—Ag3	75.24 (11)	C29—C30—C26	119.0 (5)
Ag7—Ag6—Ag3	80.29 (2)	C29—C30—H30A	120.5
O10—Ag7—O8	161.65 (15)	C26—C30—H30A	120.5
O10—Ag7—Ag6	81.73 (11)	O11—C31—O12	125.2 (5)
O8—Ag7—Ag6	79.97 (11)	O11—C31—C32	119.3 (5)
O10—Ag7—Ag8	73.02 (11)	O12—C31—C32	115.5 (5)
O8—Ag7—Ag8	125.32 (11)	C36—C32—C33	118.1 (5)
Ag6—Ag7—Ag8	154.36 (2)	C36—C32—C31	121.8 (5)
O10—Ag7—Ag4	114.52 (13)	C33—C32—C31	120.1 (5)
O8—Ag7—Ag4	67.86 (12)	C34—C33—C32	119.4 (5)
Ag6—Ag7—Ag4	100.15 (3)	C34—C33—H33A	120.3
Ag8—Ag7—Ag4	94.38 (2)	C32—C33—H33A	120.3
N6^iii^—Ag8—O12	171.76 (17)	N6—C34—C33	122.5 (5)
N6^iii^—Ag8—Ag7	125.28 (13)	N6—C34—H34A	118.7
O12—Ag8—Ag7	56.90 (11)	C33—C34—H34A	118.7
N1—C1—C2	123.9 (5)	N6—C35—C36	122.6 (5)
N1—C1—H1A	118.0	N6—C35—H35A	118.7
C2—C1—H1A	118.0	C36—C35—H35A	118.7
C1—C2—C3	118.6 (5)	C32—C36—C35	119.5 (5)
C1—C2—H2A	120.7	C32—C36—H36A	120.3
C3—C2—H2A	120.7	C35—C36—H36A	120.3
C4—C3—C2	117.7 (5)	C5—N1—C1	117.3 (5)
C4—C3—C6	120.8 (5)	C5—N1—Ag1	120.0 (4)
C2—C3—C6	121.4 (5)	C1—N1—Ag1	122.6 (4)
C3—C4—C5	120.3 (5)	C11—N2—C10	116.1 (5)
C3—C4—H4A	119.9	C11—N2—Ag1^iv^	122.0 (4)
C5—C4—H4A	119.9	C10—N2—Ag1^iv^	121.9 (3)
N1—C5—C4	122.1 (5)	C16—N3—C17	116.8 (5)
N1—C5—H5A	118.9	C16—N3—Ag2^v^	119.1 (4)
C4—C5—H5A	118.9	C17—N3—Ag2^v^	124.0 (4)
O2—C6—O1	126.4 (5)	C23—N4—C19	117.1 (5)
O2—C6—C3	117.7 (5)	C23—N4—Ag5	121.8 (3)
O1—C6—C3	115.9 (5)	C19—N4—Ag5	121.0 (4)
O3—C7—O4	126.8 (5)	C28—N5—C29	117.3 (5)
O3—C7—C8	115.6 (5)	C28—N5—Ag5^iv^	120.3 (4)
O4—C7—C8	117.6 (5)	C29—N5—Ag5^iv^	122.4 (4)
C12—C8—C9	116.7 (5)	C35—N6—C34	117.9 (5)
C12—C8—C7	121.2 (5)	C35—N6—Ag8^vi^	125.5 (4)
C9—C8—C7	122.1 (5)	C34—N6—Ag8^vi^	116.6 (4)
C10—C9—C8	119.5 (5)	O14—N7—O15	119.9 (5)
C10—C9—H9A	120.2	O14—N7—O13	120.4 (5)
C8—C9—H9A	120.2	O15—N7—O13	119.6 (5)
N2—C10—C9	123.9 (5)	O17—N8—O16	120.7 (5)
N2—C10—H10A	118.1	O17—N8—O18	120.2 (6)
C9—C10—H10A	118.1	O16—N8—O18	119.0 (5)
N2—C11—C12	123.6 (5)	C6—O1—Ag3	124.3 (4)
N2—C11—H11A	118.2	C6—O2—Ag4	123.7 (4)
C12—C11—H11A	118.2	C7—O3—Ag3	126.4 (4)
C11—C12—C8	120.2 (5)	C7—O4—Ag4	121.6 (4)
C11—C12—H12A	119.9	C13—O5—Ag2	118.1 (4)
C8—C12—H12A	119.9	C13—O5—Ag3	110.0 (3)
O6—C13—O5	125.9 (6)	Ag2—O5—Ag3	90.39 (14)
O6—C13—C14	118.9 (5)	C13—O6—Ag6	114.5 (3)
O5—C13—C14	115.1 (5)	C24—O7—Ag6	121.5 (4)
C18—C14—C15	118.8 (5)	C24—O8—Ag7	126.8 (4)
C18—C14—C13	122.0 (5)	C25—O9—Ag6	122.5 (4)
C15—C14—C13	119.2 (5)	C25—O10—Ag7	124.7 (4)
C16—C15—C14	119.3 (5)	C31—O11—Ag4	123.8 (4)
C16—C15—H15A	120.3	C31—O12—Ag8	113.9 (4)
C14—C15—H15A	120.3		

Symmetry codes: (i) *x*, *y*−1, *z*; (ii) −*x*+1, *y*−1/2, −*z*+1/2; (iii) −*x*+2, *y*+1/2, −*z*+3/2; (iv) *x*, *y*+1, *z*; (v) −*x*+1, *y*+1/2, −*z*+1/2; (vi) −*x*+2, *y*−1/2, −*z*+3/2.
